# Advanced Glycation Endproducts Interfere with Adhesion and Neurite Outgrowth

**DOI:** 10.1371/journal.pone.0112115

**Published:** 2014-11-11

**Authors:** Dorit Bennmann, Rüdiger Horstkorte, Britt Hofmann, Kathleen Jacobs, Alexander Navarrete-Santos, Andreas Simm, Kaya Bork, Vinayaga S. Gnanapragassam

**Affiliations:** 1 Institute for Physiological Chemistry, Martin-Luther-University Halle-Wittenberg, Halle (Saale), Germany; 2 Department of Cardiothoracic Surgery, University Hospital, Halle (Saale), Germany; Queen's University Belfast, United Kingdom

## Abstract

Advanced glycation endproducts (AGEs) represent a non-enzymatic posttranslational protein modification. AGEs are generated by a series of chemical reactions of free reducing monosaccharides, such as glucose, fructose or metabolites of the monosaccharide metabolism with amino groups of proteins. After oxidation, dehydration and condensation, stable AGE-modifications are formed. AGE-modified proteins accumulate in all cells and tissues as a normal feature of ageing and correlate with the glucose concentration in the blood. AGEs are increased in diabetic patients and play a significant role in the pathogenesis of most age-related neural disorders, such as Alzheimer’s disease. We examined the role of AGEs on neurite outgrowth of PC12 cells. We induced the formation of AGEs using the reactive carbonyl compound methylglyoxal (MGO) as a physiological metabolite of glucose. We found that AGE-modification of laminin or collagen interfered with adhesion but not with neurite outgrowth of PC12 cells. Furthermore, the AGE-modification of PC12 cell proteins reduced NGF-induced neurite outgrowth. In conclusion, our data show that AGEs negatively influence neural plasticity.

## Introduction

**Figure 1:**
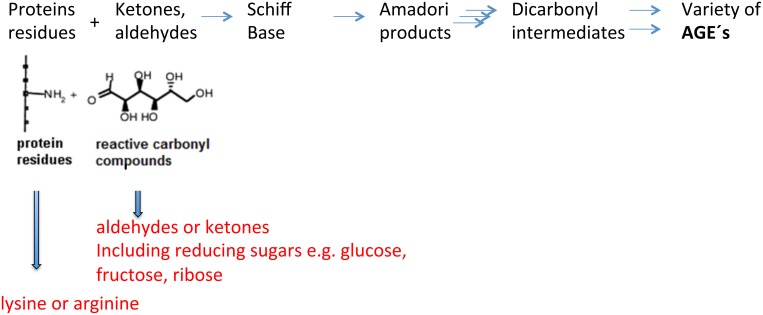
Formation of advanced glycation endproducts (AGE).

More than 100 years ago Louis Camille Maillard discovered the essential reaction that leads to the formation of advanced glycation endproducts (AGEs). The initial reaction for the formation of AGEs is a non-enzymatic glycosylation (glycation) also known as Mailliard reaction, at the ε-amino group of lysine or at its free amino group [1], [2]. In addition, side chains of the amino acids cysteine, arginine or tryptophan are also potential sites for glycation. The resulting Schiff base adducts rearrange to so-called Amadori products. After further oxidation and dehydration reactions that include the formation of radical intermediates, fluorescent and yellow–brown covalently cross-linked AGEs are generated (Fig. 1). The accumulation of reactive carbonyl precursors or glycooxidation products is termed carbonyl stress. Frequent and well-characterized AGEs are carboxymethyllysine (CML), carboxyethyllysine (CEL) and pentosidine or non-oxidative AGEs such as glyoxal lysine dimer (GOLD), methylglyoxol lysine dimer (MOLD), desoxyglucasone lysine (DOLD) or pyrroline [3]. CML and CEL can be detected by very specific antibodies and serve as biomarkers for oxidative stress and ageing [4], [5].

The history of research on AGEs in association to age-related diseases started with the detection of glycated hemoglobin in the 1970s. In the 1980s, Monnier and Cerami, the pioneers of the non-enzymatic glycosylation theory of ageing, proposed that AGE-modifications are responsible for the “malfunction” of proteins by participating in cellular ageing [6], [7]. AGEs accumulate over time and are used as markers of carbonyl stress [8]. Advanced glycation endproducts are very stable and protease-resistant. Therefore, AGE-induced crosslinks of peptides and proteins lead to protein deposition and amyloidosis, which is a reason why AGEs are involved in a variety of diseases, such as arteriosclerosis, diabetic nephropathy and neuropathy or cataract [9]. As an example, AGEs have been detected in vascular walls, glomerular basement membranes and the renal cortex, as well as in amyloid plaques in Alzheimeŕs disease. During AGE formation, oxygen radicals are also generated, which, beside the AGEs themselves, are involved in neuronal cell damage by oxygen stress and apoptotic processes [10], [11]. Taken together, the glycation theory is one explanation for the molecular mechanism of ageing. In line with this theory, crosslinking and denaturation of proteins caused by glycation, are major factors for early ageing [12], [13]. However, glycation of proteins is not the only mechanism of ageing. Oxidative damage by reactive oxygen species is also involved in ageing. This implies that every cell is under constant pressure to remove waste that accumulates in form of metabolically damaged proteins and xenobiotics. Preventing the generating of these metabolic waste products help to prevent cellular ageing and has been termed the “green theory” of ageing (for review see: [14]). In this study we focused on the involvement of AGEs on neuronal adhesion and differentiation because age-related accumulation of AGEs has been shown in different regions of the human brain [15], [16]. We could demonstrate that AGE-modified substrates (e.g., extracellular matrix components) interfere with cell adhesion of PC12 cells. Furthermore, we found that moderate AGE-modifications of the cell surfaces did not influence cell viability in culture but strongly interfered with NGF-induced neurite outgrowth by preventing extracellular signal-regulated kinase (ERK) phosphorylation.

## Methods

### Cell culture

Rat adrenal medulla pheochromocytoma cells (PC12) [16] were cultured in RPMI-1640 with 5% heat-inactivated fetal calf serum (FCS), 10% horse serum (HS) and 2 mM L-glutamine in humidified 5% CO_2_ atmosphere at 37°C. The cultures were also supplemented with 100 U/ml penicillin and 100 µg/ml streptomycin (all PAA, Cölbe, Germany).

### Cellular AGE-formation

When cells reached 80% confluence, the reactive carbonyl compound methylglyoxal (MGO) (Sigma-Aldrich, St. Louis, USA) was added at different final concentrations of 0.1 mM, 0.3 mM or 1.0 mM to serum-free cell culture media for 4 hours in a humidified 5% CO_2_ atmosphere at 37°C. Untreated PC12 cells cultured under serum-free conditions were used as control. After the incubation time, cells were harvested, centrifuged at 1100 rpm for three minutes and washed twice with sterile PBS (pH 7.4) or were cultured under serum containing conditions.

### Matrix AGE-modification

Cell culture plates were coated with collagen IV or laminin (20 µg/ml for 2 hours; OMNI LIFE Science, Bremen, Germany). Plates were AGE-modified with 1.0 mM MGO (Sigma-Aldrich, St. Louis, USA) for 4 hours at 37°C. Some plates were divided into two parts and only one half of the plates was AGE-modified as above and thoroughly washed with PBS.

### Cell viability by tryphan blue staining

PC12 cells were incubated with MGO as described. Immediately after the incubation, cells were centrifuged at 1100 rpm for 3 minutes and washed with PBS (pH = 7.4). Cells were re-suspended in PBS and mixed 1∶1 with trypan blue dye (0.4%; Invitrogen, Darmstadt, Germany). Cells were counted using an automated cell counter (Countess; Invitrogen, Darmstadt, Germany).

### Analytical procedures

Protein concentrations were determined using a 25 µl sample in 96-well ELISA plates with 200 µl bicinchoninic acid protein reagent (Pierce, Thermo Fisher Scientific, Rockford, USA). Plates were evaluated in a 96-well ELISA reader (Thermo Fisher Scientific, Rockford, USA) at 560 nm.

### Preparation of cell extracts

Cell pellets were solubilized at 4°C for one hour in buffer containing 150 mM NaCl, 50 mM Tris, 1 mM CaCl_2_, 1 mM MgCl_2_, 1% Triton, and protease inhibitor cocktail (Sigma-Aldrich, St. Louis, USA) at pH 7.4. After centrifugation at 13000 rpm for 10 minutes, supernatants were collected.

### Cell adhesion assay

A real-time cell analyzer (RTCA) (OMNI Life Science, Bremen, Germany) was used to quantify cell adhesion. E plates were coated with 20 µg/ml laminin or collagen IV in PBS for 1 hour at 37°C. After coating, the E plates were washed with PBS and blocked with 0.5% BSA for 30 minutes at room temperature. After two further washings with PBS, 0.5×10^5^ PC12 cells were seeded onto coated E plates and cell adhesion was continuously monitored for 4 hours.

### Immunoblotting

Samples were separated on SDS-polyacrylamide gels (CBS, Scientific, Germany) and transferred to nitrocellulose membrane. The blots were blocked with 3% gelatin in PBS. AGE formation was detected using a lysine-metabolized AGE-specific monoclonal antibody CML at a concentration of 0.1 µg/ml (Abcam, Cambridge, England). For the detection of the NGF-induced activated pathway, antibodies to ERK1/2 and phospho-ERK1/2 (Abcam, Cambridge, England) were used at a concentration of 0.1 µg/ml. Proteins were detected by enhanced chemiluminescence (Pierce, Thermo Fisher Scientific, Rockford, USA) according to the manufacturer’s instructions and visualized by exposing blots for 10 to 120 seconds using a a BioRad Imager system (BioRad, München, Germany.

### Neurite outgrowth

Neurite outgrowth was determined as described in Pollscheit et al. [18]. In brief, 5×10^3^ PC12 cells were seeded on laminin coated (20 µg/ml) E-plates (OMNI Life Science, Bremen, Germany). Cells were cultured for 48 hours in the absence or presence of 100 ng/ml NGF (ImmunoTools, Friesoythe, Germany). Neurite outgrowth of the cultures was continuously analyzed by the xCELLigence system (OMNI Life Science, Bremen, Germany). After 48 hours, cultures were fixed with 4% paraformaldehyde in PBS for 10 minutes, stained with crystal violet, and micrographs were taken as visual controls.

### Detection of AGE-modification by flow cytometry

5×10^6^ AGE-modified PC12 cells were fixed by 4% paraformaldehyde/PBS for 10 minutes at 4°C and permeabilized with 0.1% PBS-tween for 2 minutes at 4°C. Cells were then blocked with PBS/0.3 M glycine/5% FCS for 15 minutes at 4°C. CML antibody was added at a concentration of 0.5 µg/ml for one hour at 4°C before incubating with the Delight 488 - fluorescence labeled secondary anti-mouse antibody (Invitrogen, Darmstadt, Germany) for one hour at 4°C. Cells were washed and re-suspended in PBS and analyzed in an Accuri C6 flow cytometer (BD Biosciences, Heidelberg, Germany).

### Apoptosis assay

5×10^6^ PC12 cells were labeled with annexin V-FITC and propidium iodide (PI) (Apoptosis Detection Kit, abcam, Cambridge, England). Cells were analyzed using a dual-laser Accuri C6 flow cytometer (BD, Heidelberg, Germany). Annexin V-FITC and PI signals were excited using a 488-nm laser light. For dual labeling, fluorescence emissions of individual fluorophores were corrected for spectral overlay using electronic compensation.

## Results

### Cell adhesion but not neurite outgrowth of PC12 cells is reduced on AGE-modified ECM proteins

**Figure 2:**
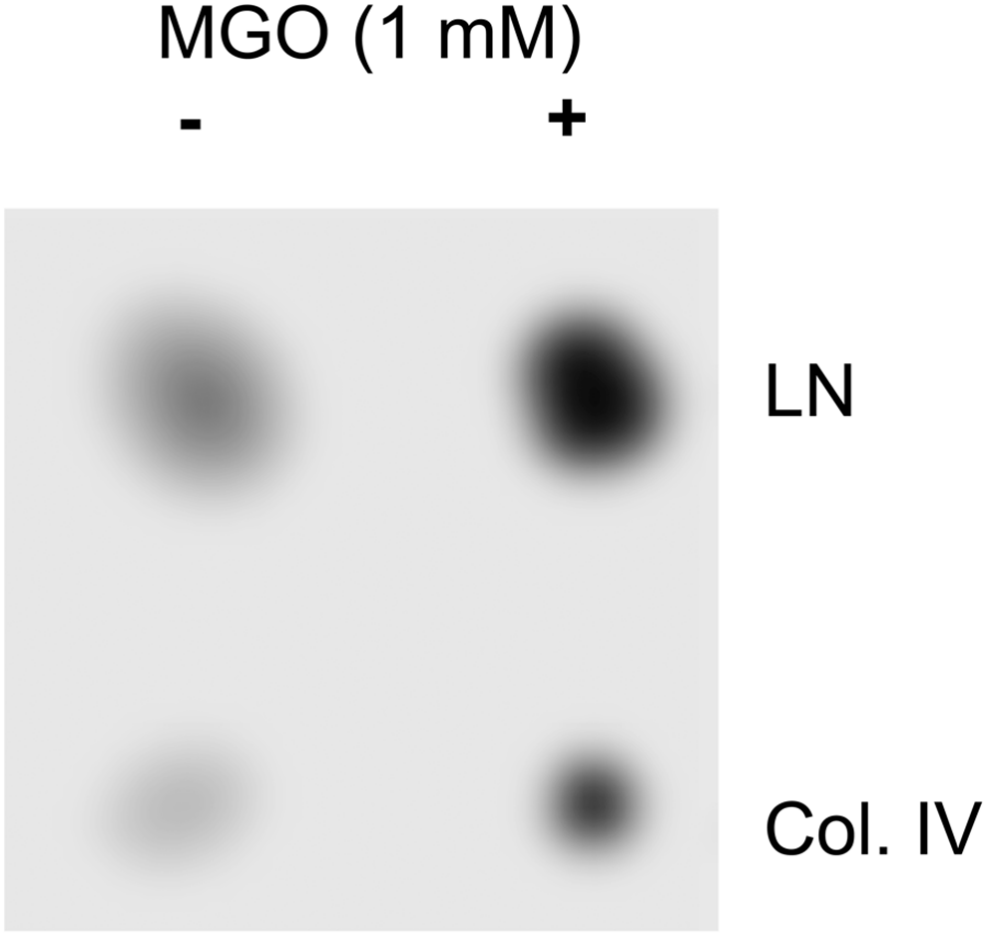
AGE modification of ECM proteins using MGO. 20 µg collagen IV (Col. IV) or laminin (LN) were spotted on a nitrocellulose membrane. One half of the membrane was incubated with 1 mM MGO for 4 hours. AGE formation was detected by dot blot analysis using the monoclonal CML26 antibody.

**Figure 3:**
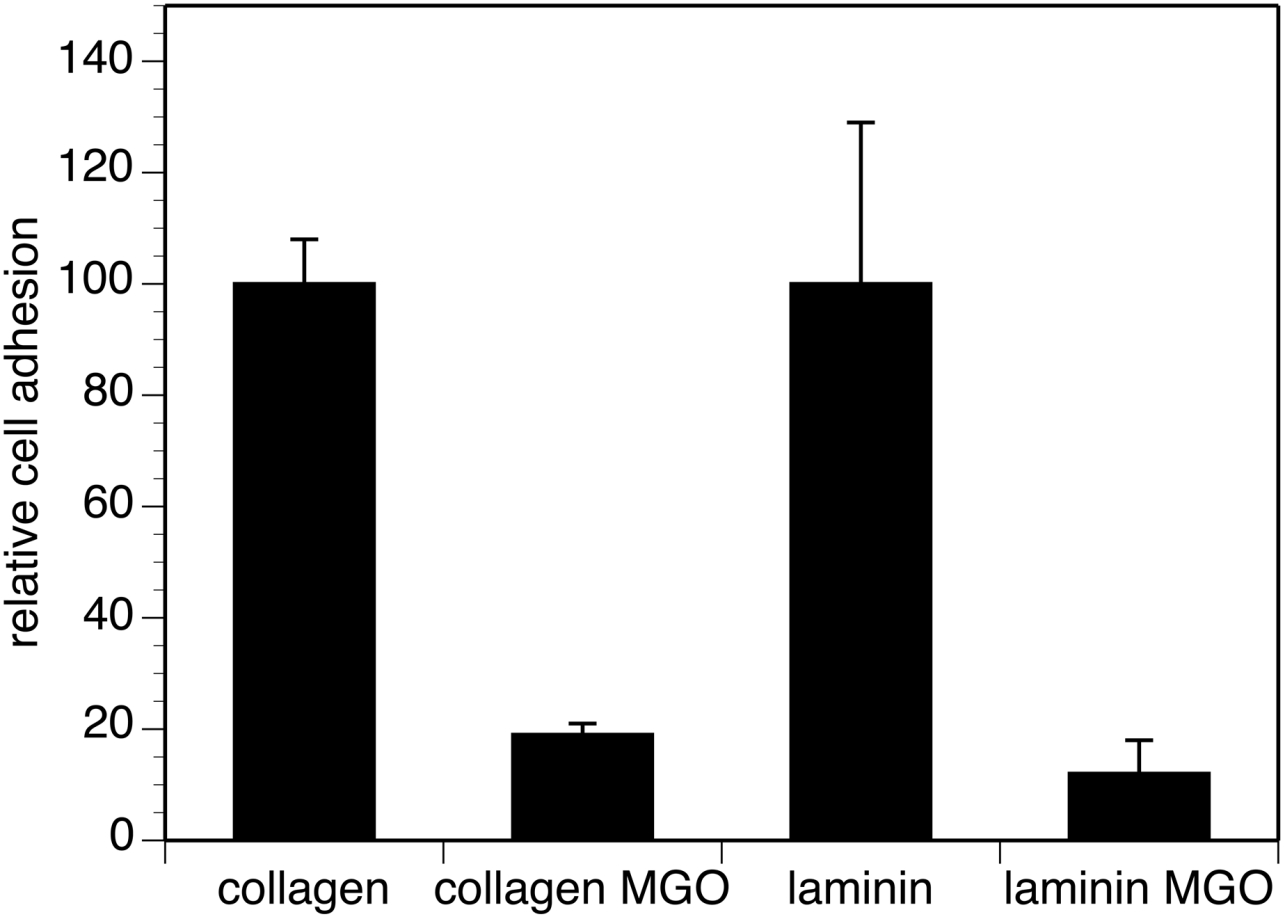
Cell adhesion of PC12 cells to AGE-modified ECM proteins. AGE-modified and non-modified collagen IV and laminin (shown in Fig. 2) were coated on E-plates. Cell adhesion of 5×10^5^ PC12 cells was quantified by RTCA real time analysis as described. Adhesion to non-modified ( = control) substrates was set to 100% and adhesion to AGE-modified substrates was calculated in % of control. Each bar represents values of three independent experiments carried out in triplicates (*p≤0.0001).

**Figure 4:**
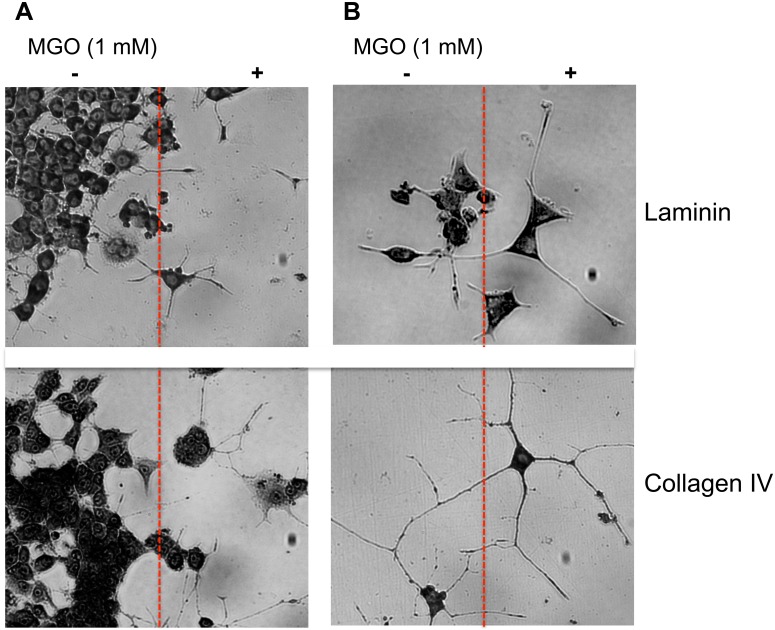
Cell adhesion and neurite outgrowth at the border of AGE-modified ECM proteins. A. Representative micrographs of PC12 cells taken at the border (red line) of AGE-modified or non-AGE-modified laminin or collagen IV coated cell culture plates. Three independent experiments were performed. Note that PC12 cells prefer non-AGE-modified laminin or collagen IV. B. Representative micrographs of neurite outgrowth at the border of AGE-modified or non-AGE-modified laminin or collagen IV coated cell culture plates. Three independent experiments were performed. Note that neurite outgrowth of PC12 cells was not different between non-AGE-modified laminin or collagen IV or AGE-modified laminin and collagen IV.

Cell adhesion is a crucial step for neurite outgrowth and regeneration. We compared cell adhesion of PC12 cells on unmodified or AGE-modified laminin or collagen IV. First we checked the AGE-modification by dot blot analysis (Fig. 2). We were able to induce AGE-modifications on laminin and collagen IV with MGO. We then quantified cell adhesion of PC12 cells on these AGE-modified ECM proteins by real-time analysis. We found that AGE-modification of collagen resulted in an 80% reduction of cell adhesion and a reduction of cell adhesion on laminin of approximately 90% (Fig. 3). We visually analyzed PC12 cells grown on AGE-modified and non- AGE-modified laminin or collagen IV in the absence or presence of NGF (Fig. 4). We found fewer cells on the AGE-modified matrices compared to non-AGE-modified matrices (Fig. 4A), which confirms the reduction in cell adhesion on AGE-modified laminin or collagen IV shown in figure 3. Interestingly, we observed no differences in neurite outgrowth at the borders of AGE-modified and non-AGE-modified laminin or collagen IV (Fig. 4B).

### AGE-modification of PC12 cells does not influence their viability

**Figure 5:**
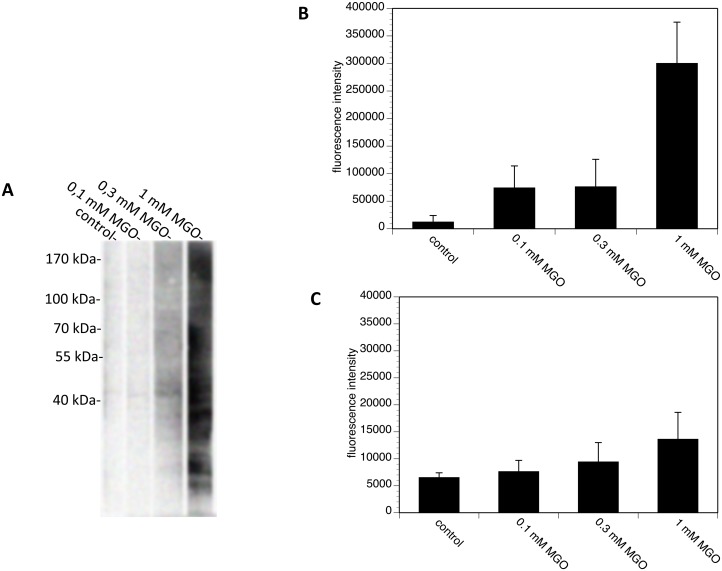
AGE-modification of PC12 cells using MGO. PC12 cells were incubated with PBS, 0.1 mM MGO, 0.3 mM MGO or 1.0 mM MGO for 4 hours. A. Washed cells were solubilized and subjected to SDS-gel electrophoresis. Proteins were blotted and detected using monoclonal CML26 antibody B&C. Permeabilized (B) and non-permeabilized (C) were analyzed by flow cytometry using monoclonal CML26 antibody.

**Figure 6:**
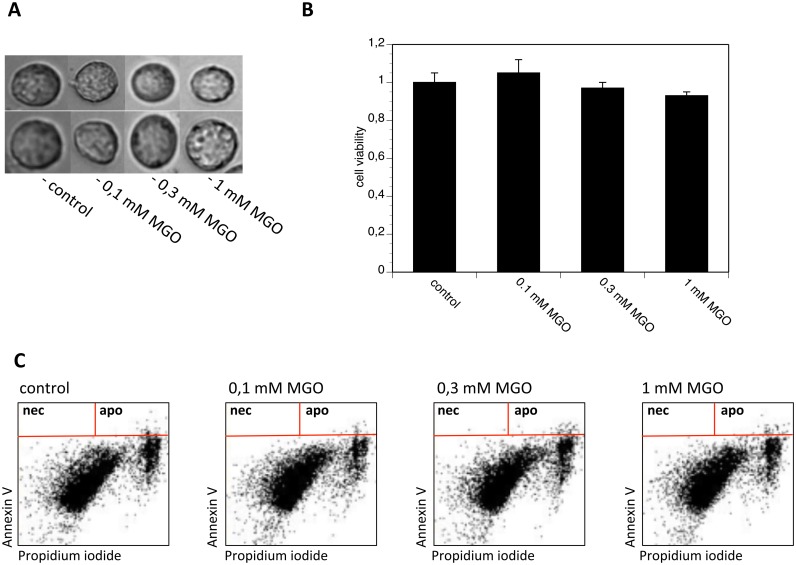
Cell viability after MGO treatment. PC12 cells were incubated with PBS, 0.1 mM MGO, 0.3 mM MGO or 1 mM MGO for 4 hours. A. Micrographs of typical PC12 cells. B. Tryphan blue staining of PC12 cells. Bars represent three independent experiments carried out in quadruplicates. Cell viability of cells cultured in the presence of PBS ( = control) was set to 1 and cell viability expressed in relation to the control. C. FACS analysis of PC12 cells stained with annexin V and propidium iodide.

Since not only matrix proteins but also cell surface proteins can be modified by AGEs, we next tried to establish a method to AGE-modify cellular proteins without interfering with cell viability. We incubated PC12 cells in the presence of serum-free medium with several concentrations of MGO and quantified the formation of AGE by Western blot or FACS analysis (Fig. 5A). Application of 0.1 mM MGO did not lead to the formation of AGE on cellular proteins, whereas after application of 1 mM MGO, many cellular proteins showed AGE-modification as indicated by a broad smear in the Western blot analysis (Fig. 5A). This could be confirmed by FACS analysis (Fig. 5B). In order to verify AGE-modification after MGO treatment on the cell surface (as MGO is cell permeable), we performed FACS analysis of non-permeabilized cells. Figure 5C summarizes the results, indicating that cell surface proteins are AGE-modified after 1 mM MGO application. Based on the Western blot analysis, it appeared that a larger amount of intracellular protein than surface protein was modified by MGO treatment (compare Fig. 5B with C). We then compared micrographs of PC12 cells that were treated with 0.1 mM, 0.3 mM or 1 mM MGO (Fig. 6A). MGO treatment of PC12 cells had no obvious effect on cell morphology. Furthermore, we performed a trypan blue exclusion assay and confirmed the observation, that the cell viability of MGO-treated cells was comparable to untreated cells (Fig. 6B).

One early event during apoptosis is the transition of phosphatidyl-serine from the inner to the outer membrane facing the cell surface. Phosphatidyl-serine can be detected by the Ca2+-dependent phosphatidyl-binding protein annexin V. To quantify the potential loss of membrane integrity, we labeled PC12 cells with annexin V-FITC and stained with propidium iodide, which stains cells with compromised membrane integrity. We did not observe any obvious difference between untreated or MGO-treated PC12 cells (Fig. 6C).

### AGE-modifications interfere with neurite outgrowth

**Figure 7:**
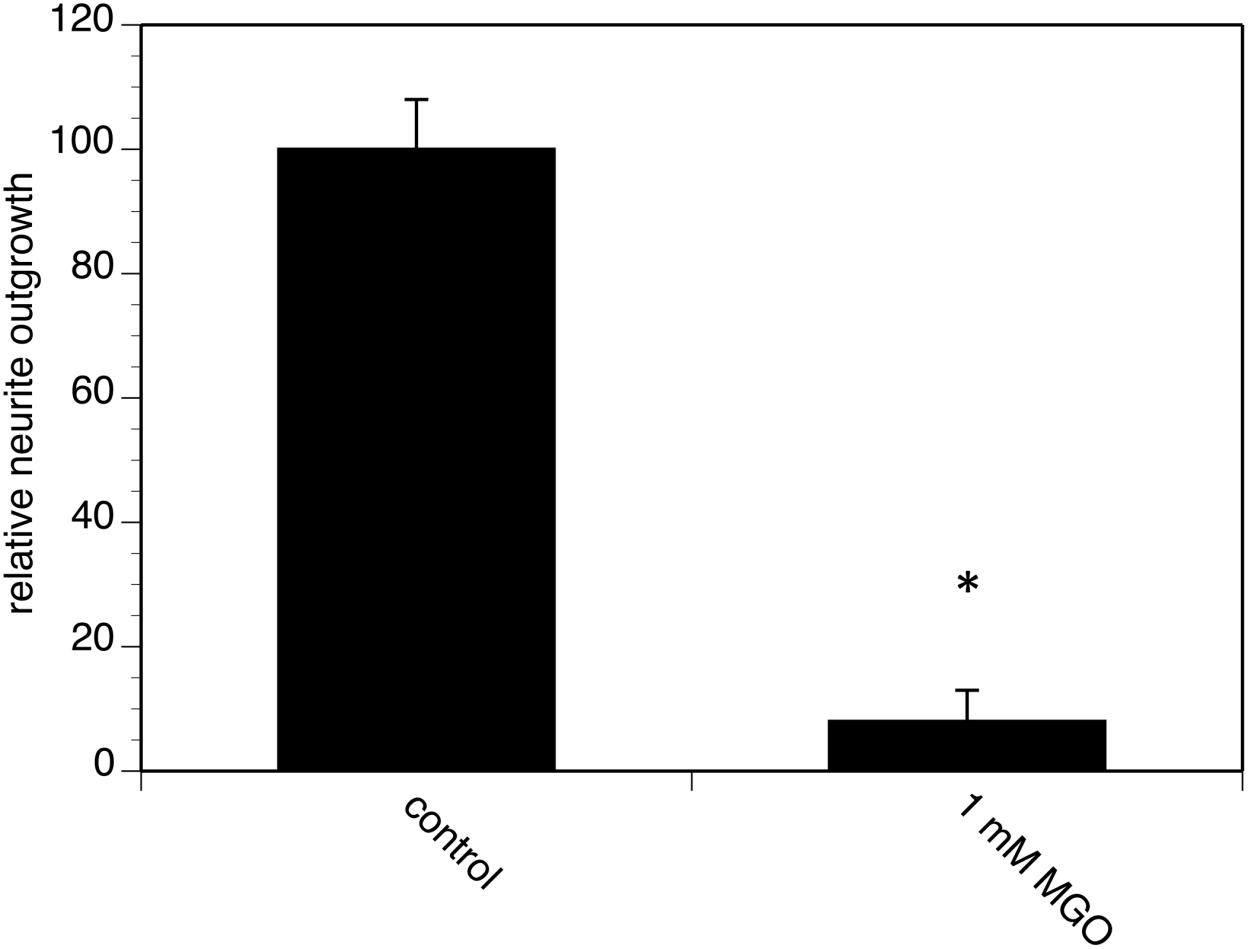
Real-time analysis of neurite outgrowth of AGE-modified PC12 cells. PC12 cells were AGE-modified using 1 mM MGO for 4 h as shown in Figs. 4 &5. Cells were cultured on LN-coated E-plates and neurite outgrowth was induced by application of 100 ng/ml NGF. Neurite outgrowth was continuously quantified over 48 hours by RTCA as described in Pollscheit et al. (2012). Total neurite outgrowth of non-modified control cells during 48 h was set to 100% and neurite outgrowth of AGE-modified cells was expressed in % of control. Bars represent two independent experiments carried out in quadruplicates.

Regenerating capacity of neurons decreases with age [19]. To answer the question whether AGEs might be involved in this process, we quantified neurite outgrowth of AGE-modified PC12 cells compared to non-AGE-modified cells. PC12 cells are a well-known cellular system to study neurite outgrowth as model for regeneration [17]. We cultured non-AGE-modified PC12 cells in the presence of nerve growth factor (NGF) and found prominent neurite outgrowth. However, PC12 cells incubated with 1 mM MGO showed a dramatic decrease of neurite outgrowth, resulting in a reduction of more than 90% (Fig. 7). This inhibition of neurite outgrowth by AGEs was dose-dependent (data not shown).

**Figure 8:**
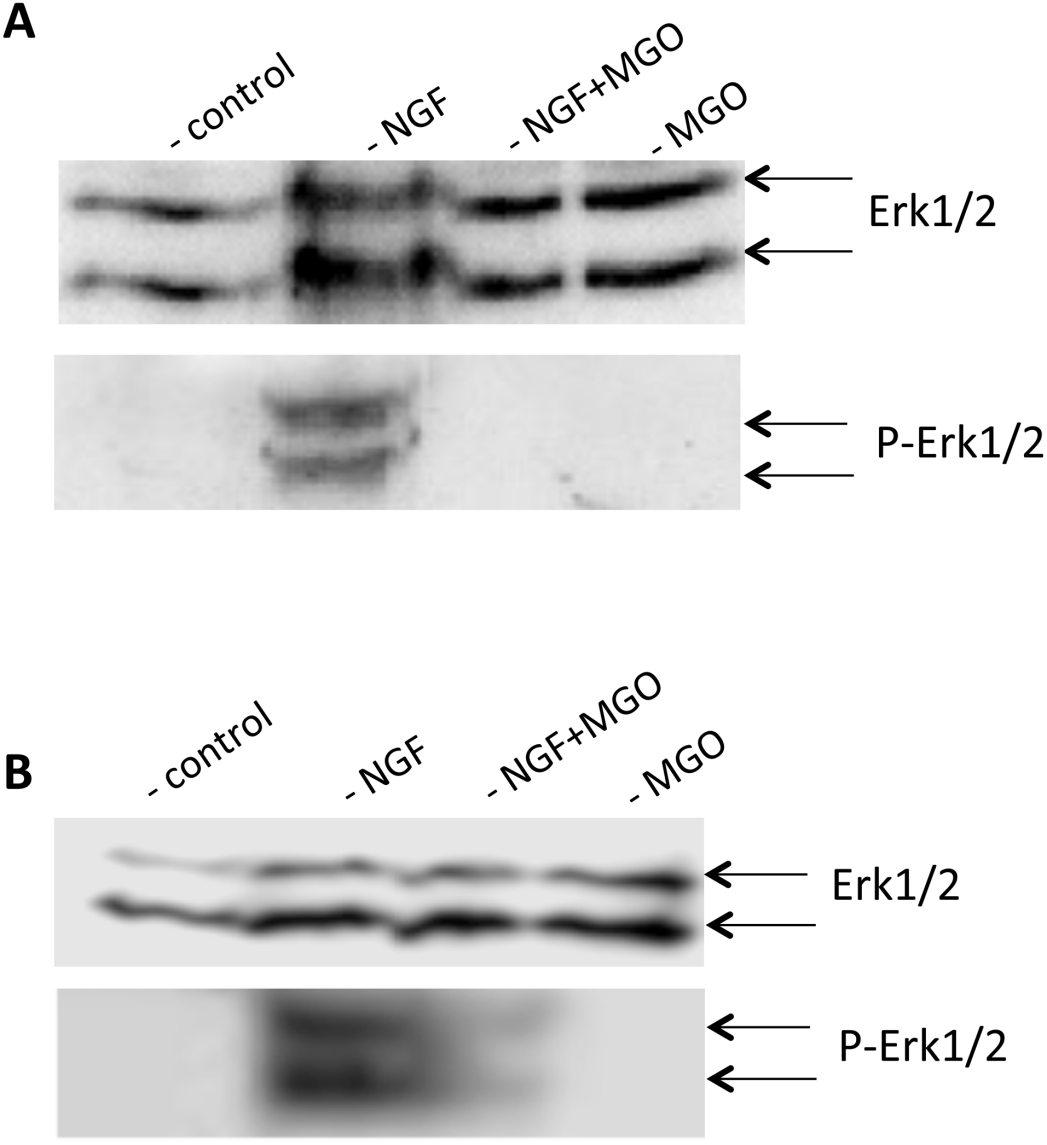
ERK1/2 activation of AGE-modified PC12 cells. PC12 cells were incubated with PBS or 1 mM MGO for 4 hours and stimulated with 100 ng/ml NGF. A. Cells were harvested, washed, and solubilized. Solubilizates were centrifuged and subjected to SDS-gel electrophoresis. Proteins were blotted and detected using monoclonal ERK1/2 antibody for equal loading and monoclonal phospho ERK1/2 antibody for the detection of ERK activation. B. After MGO-treatment, cells were cultured for further 72 hours, harvested, washed, and solubilized. Solubilizates were centrifuged and subjected to SDS-gel electrophoresis. Proteins were blotted and detected using monoclonal ERK1/2 antibody for equal loading and monoclonal phospho-ERK1/2 antibody for the detection of ERK activation.

NGF binds on TRK A-receptors and activates the intracellular MAPK pathway, which is responsible for neurite outgrowth. To elucidate the underlying molecular effects of MGO-mediated AGE-formation on NGF signal transduction pathways, we performed Western blots for ERK1/2 and its activated form phospho-ERK1/2. NGF-treated PC12 cells served as positive control as described by Kontou et al. [20]. All PC12 cultures expressed ERK1/2 (Fig. 8A and B, upper blots) and we detected active phospho-ERK1/2 in cells cultured in the presence of NGF (Fig. 8A lower blot). As expected, we found no active phospho-ERK1/2 in MGO-treated cells cultured in the presence of NGF. However, when analyzing phosphorylation of ERK1/2 72 h after the induction of glycation by MGO, we found phosphorylated ERK1/2 (Fig. 8B bottom blot), which indicates that cells recovered from MGO-induced glycation stress.

## Discussion

Ageing is associated with decreased regenerative capacity of the nervous system. One prominent molecular event during ageing is the accumulation of advanced glycation endproducts (AGEs). Accumulation of AGEs has been identified in different compartments and regions in the human brain in an age-related manner [15], [16]. Long-living proteins (e.g. extracellular matrix proteins) serve as ideal substrates for the glycation reactions and therefore AGE formation occurs even in the presence of physiological concentrations of glucose or fructose over a long time. In this study, we focused on the effect of AGE-accumulation for neuronal cells. We induced AGE-formation by methylglyoxal (MGO), which occurs during normal catabolism of glucose. Note that the brain, e.g., neurons depends on glucose as energy source and shows a high rate of glycolysis. MGO-induced AGE-formation is an accepted agent to stimulate age-dependent disorders [21]. We found that AGE-modifications of extracellular matrix proteins interfered with cell adhesion of PC12 cells. Note that long-lived proteins, such as the ECM components laminin and collagen, serve as accumulated substrates for AGE-modification [22]. Since regenerating neurons or axons need contact to these proteins [23], our data on cell adhesion to AGE-modified ECM proteins explain the reduced regenerating capacity in an AGE-modified environment. Interestingly, AGE-modifications of PC12 cells had no effect on their viability (when using 1 mM MGO in the presence of serum-free media, considering that higher concentrations of MGO are toxic). We further quantified neurite outgrowth by using nerve growth factor (NGF) since it is well known that NGF-treated PC12 cells stop proliferation, extend neurites and become electrically excitable [17]. We found that AGE-modified PC12 cells extended nearly no neurites in the presence of NGF. However, AGE-modification at the border of the underlying substrate (e.g. laminin or collagen IV) had no effect on border-crossing neurite outgrowth. NGF binds to TRK receptors [24] and activates the MAP kinase pathway [25] or the PLCγ/PKC pathway [26], which inhibits proliferation and initiates neurite outgrowth. We could demonstrate that AGE-modified PC12 cells did not respond to NGF, while untreated PC12 responded to NGF by activation of the ERK1/2 kinases followed by neurite outgrowth as described by Tomaselli et al. [27] or Pollscheit et al. [18].

We therefore assume that AGE-formation of the extracellular matrix has only small effects (reduced cell adhesion) but that cellular AGE-formation has a large influence on cell signaling processes in (neuronal−) cells. Disturbed cell signaling pathways could lead to an imbalance in the cellular signal transduction pathways and finally to impaired function.

There could be another explanation for the death of nerve cells during Alzheimer diseases. Morbus Alzheimer is not only characterized by reduction of nerve cells in the hippocampus and neocortex region; it is also accompanied by increasing levels of extracellular β-amyloid plaques, which leads us to another interesting aspect. Tissues that were starved or show low regeneration ability, such as altered brain tissue, cannot handle accumulation of modified intracellular proteins [28]. The irreversible non-enzymatic glycosylation of reactive amino acids of proteins that lead to AGE formation modulates protein properties such as stability, conformation or folding. AGE-modifications could lead to restrictions of protein functions, reduction or increase of half-life time followed by accumulation. This is of special interest since AGE-modified proteins had been already detected in ß-amyloid plaques of Alzheimer patients [29].

Taken together, we could demonstrate that AGE-modifications of neuronal cells have striking consequences. AGEs interfere with cell adhesion and reduce neurite outgrowth dramatically. Our results indicate, that “protein-AGEing” interferes with cell adhesion, cell signaling and protein function in general and we conclude that AGEs are negative prognostic markers for neural plasticity.
